# A contextual behavioral model of chemsex: structural equation modeling of psychological predictors of hypersexuality and drug use

**DOI:** 10.3389/fpsyg.2025.1672471

**Published:** 2025-11-18

**Authors:** Rubén Rico, Francisco Montesinos

**Affiliations:** Departamento de Psicología, Facultad de Ciencias Biomédicas y de la Salud, Universidad Europea de Madrid, Madrid, Spain

**Keywords:** chemsex, psychological flexibility, sexualized drug use, hypersexuality, structural equation modeling

## Abstract

**Background and objective:**

Chemsex, defined as the intentional use of psychoactive substances in sexual contexts, is increasingly prevalent among gay and bisexual men and is associated with a range of mental, physical and social implications. This study aimed to test a contextual behavioral model examining how psychological inflexibility, loneliness, intimacy difficulties, and internalized homophobia contribute to hypersexuality and sexualized substance use.

**Methods:**

A cross-sectional study was conducted with 252 Spanish-speaking participants who reported engaging in sexualized substance use within the past year. Standardized self-report measures were used to assess psychological processes and behavioral outcomes. Structural equation modeling was employed to examine direct and indirect effects among variables.

**Results:**

The proposed path model demonstrated excellent fit (*χ*^2^ = 0.002, *p* = 0.967; CFI = 1.000; RMSEA = 0.000). Psychological inflexibility showed the strongest association with both hypersexuality (*β* = 0.48, *p* < 0.001) and substance use (*β* = 0.25, *p* < 0.01), with a significant indirect effect via hypersexuality. Loneliness was also associated with both outcomes and showed mediated effects. Internalized homophobia was associated with hypersexuality but not with substance use. Intimacy was not a significant predictor. The model explained 42.1% of the variance in hypersexuality and 27.2% in substance use.

**Conclusion:**

This study offers a novel contribution by modeling chemsex-related behaviors as forms of experiential avoidance shaped by psychological inflexibility, loneliness, and internalized homophobia. The findings highlight the potential utility of Acceptance and Commitment Therapy (ACT) in reducing chemsex vulnerability by promoting psychological flexibility and values-based living.

## 1 Introduction

The term chemsex refers to the intentional use of psychoactive substances in sexual contexts, typically aimed at enhancing or prolonging sexual activity (Esteban et al., 2024). While not exclusive to any particular demographic, this practice has been predominantly documented among gay, bisexual, and other men who have sex with men (GBMSM), as well as *trans* and non-binary individuals, especially in urban environments, and has emerged as a significant public health concern over the past decade (Bourne et al., 2015; Lafortune et al., 2021). Reported rates of chemsex among GBMSM vary widely across studies, reflecting substantial heterogeneity in definitions, populations, and measurement strategies. For example, a systematic review by Edmundson et al. (2018) found that prevalence rates over the past year ranged from 3 to 29% across European contexts. In Spain, different studies have yielded divergent prevalence estimates. Guerras et al. (2022) reported that 21.9% of their participants had engaged in chemsex in the past 12 months, while Ruiz-Robledillo et al. (2021) found a considerably higher rate of 40.6% among people living with HIV. Similarly, Íncera-Fernández et al. (2021) noted that prevalence rates across studies ranged from 10% to as high as 94%, depending on the sample and measurement criteria. Chemsex-related implications include a higher incidence of sexually transmitted infections (STIs), problematic substance use, mental health comorbidities, occupational and social impairments, and adverse effects on emotional well-being (Íncera-Fernández et al., 2021). These findings suggest that a substantial proportion of the LGBTIQ+ population engages in chemsex, particularly in urban contexts where access to psychoactive substances is greater and encounters are often facilitated through geolocation-based sexual networking applications (Hung et al., 2023). However, it is important to recognize that the potential implications of chemsex depend on multiple factors, such as frequency of use, dosage, route of administration, history of drug use, and individual psychological and social conditions. Moreover, not all GBMSM and gender-diverse individuals share the same starting point or vulnerabilities, and their substance use patterns and sexual behaviors can vary widely (Íncera-Fernández et al., 2025).

Chemsex typically entails the deliberate use of substances such as mephedrone, GHB/GBL, methamphetamine, ketamine, and cocaine, often consumed concurrently in prolonged sessions lasting several hours or days, with polydrug use being a common feature of this practice (Esteban et al., 2024; Rodríguez-Expósito et al., 2024). The practice of chemsex often facilitates disinhibition, intensify sexual sensations, alleviate social anxiety, prolong sexual encounters, often producing euphoria, increased self-confidence, and heightened libido (Lunchenkov et al., 2024; Hung et al., 2023), and is associated with significant mental health risks, including depression, anxiety, suicidal ideation, and substance dependence, with the most severe psychopathological profiles observed among individuals engaging in intravenous drug use during sex (Íncera-Fernández et al., 2021). Despite being aware of the adverse consequences of substance use, many individuals who engage in chemsex prioritize immediate perceived benefits and persist in the behavior (Lunchenkov et al., 2024), suggesting the presence of strong reinforcement mechanisms and challenges in modifying such behaviors. Chemsex may reflect a dual addictive pattern—chemical and sexual—where excessive sexual desire is both sustained and intensified by psychoactive substance use (Esteban et al., 2024; Schwarz et al., 2024). Substance use is central to chemsex, differing from conventional recreational drug use due to its intentional role in enhancing sexual experiences among GBMSM.

A psychological factor associated with chemsex is hypersexuality, also referred to as compulsive sexual behavior disorder (CSBD) or sexual addiction. It is characterized by a persistent pattern of excessive and uncontrolled sexual activity that leads to significant psychological, social, or physical consequences (Ballester-Arnal et al., 2013). In line with ICD-11, we use “Compulsive Sexual Behavior Disorder (CSBD)” when referring to the diagnostic construct and “hypersexuality” when describing elevated scores on the HBI as a dimensional indicator. We do not imply diagnosis of CSBD from HBI scores alone. A Spanish study reported that approximately 5.5% of participants met criteria for hypersexuality, with significantly higher prevalence among gay men and lesbian women compared to their heterosexual counterparts, and a generally greater occurrence among men overall (Ortega-Otero et al., 2023). Recent evidence has shown a strong association between hypersexuality, the frequency of chemsex engagement, and the number of substances consumed among GBMSM (Esteban et al., 2024). These findings suggest that chemsex may function both as a manifestation of underlying hypersexual tendencies and as a reinforcing mechanism, intensifying such behaviors through the disinhibitory effects of psychoactive substances. This heightened vulnerability may be interpreted through the lens of Minority Stress Theory (Meyer, 2003), which posits that chronic exposure to stigma, discrimination, and internalized homophobia contributes to maladaptive coping mechanisms—such as compulsive sexual behavior—as strategies for emotional regulation or identity affirmation (Pachankis et al., 2020). Within this framework, hypersexuality can be conceptualized as a behavioral response to the psychological burden imposed by hostile or invalidating social environments. The inclusion of CSBD in the ICD-11 (World Health Organization, 2019) highlights the clinical relevance of hypersexuality as a recognized mental health condition.

Loneliness and emotional intimacy deficits are central to understanding chemsex among GBMSM. Despite active social environments, many experience “paradoxical loneliness” due to social rejection, family estrangement, or difficulty forming stable bonds. Chemsex may serve as a coping mechanism, offering temporary emotional connection and a sense of belonging through drug-enhanced intimacy (Hung et al., 2023; Lafortune et al., 2021; Lunchenkov et al., 2024). However, this often leads to a cycle of emotional rebound, guilt, and continued use, reinforcing social isolation rather than alleviating it.

Internalized homophobia—defined as the internalization of negative societal attitudes toward one’s own sexual orientation or gender identity—acts as a chronic psychosocial stressor that negatively impacts mental health. It has been associated with increased levels of depression, anxiety, shame, and maladaptive coping strategies, including substance use and risky sexual behaviors (Brumfield and Dahlenburg, 2025). In the context of chemsex, internalized homophobia is a significant predictor of both initiation and persistence, contributing to psychological distress and emotional avoidance (Rodríguez-Expósito et al., 2024; Lunchenkov et al., 2024). Drug-facilitated sex may offer temporary relief from self-criticism and insecurity, but often reinforces a cycle of self-devaluation, perpetuating the belief that one’s sexuality is only acceptable under the influence of substances.

Psychological inflexibility (PI), a central concept in Acceptance and Commitment Therapy (ACT; Hayes et al., 2011), refers to the tendency to avoid or become entangled with internal experiences, leading to behavior misaligned with personal values (Hayes et al., 2006). PI has been associated with various maladaptive outcomes, including addiction and compulsive behaviors, as individuals often prioritize short-term relief over long-term goals (Krotter et al., 2024). In the context of chemsex, PI may facilitate avoidant coping strategies, such as using sex and drugs to escape distress related to loneliness, shame, or internalized homophobia. Ortega-Otero et al. (2023) found that PI was the strongest predictor of hypersexuality in a Spanish sample, surpassing other variables such as cognitive fusion and body dissociation. PI may serve as a common underlying mechanism linking various psychosocial risk factors associated with chemsex. For instance, internalized homophobia and chronic loneliness can generate persistent emotional distress. When individuals lack the flexibility to manage these experiences adaptively, they may resort to avoidant coping strategies—such as drug use and compulsive sexual behavior—that offer short-term relief but reinforce maladaptive patterns. Notably, despite the theoretical relevance of this framework, previous studies have not explicitly examined the role of PI as a transdiagnostic mechanism linking psychosocial vulnerabilities and chemsex-related behaviors.

Conversely, psychological flexibility has been identified as a protective factor against minority stress among LGBTIQ+ individuals. Higher levels of psychological flexibility are associated with reduced identity-related distress and improved psychological well-being, supporting more adaptive responses to discrimination. ACT-based interventions that promote emotional acceptance, cognitive defusion, and values-based action show promise in addressing the psychological mechanisms underlying dysfunctional chemsex engagement. Preliminary findings suggest that enhancing psychological flexibility may reduce symptoms of anxiety, depression, and compulsive sexual behavior in GBMSM, offering a transdiagnostic approach to mitigating chemsex-related harms (Montesinos et al., 2024; Chan and Yip, 2021).

**Figure 1:**
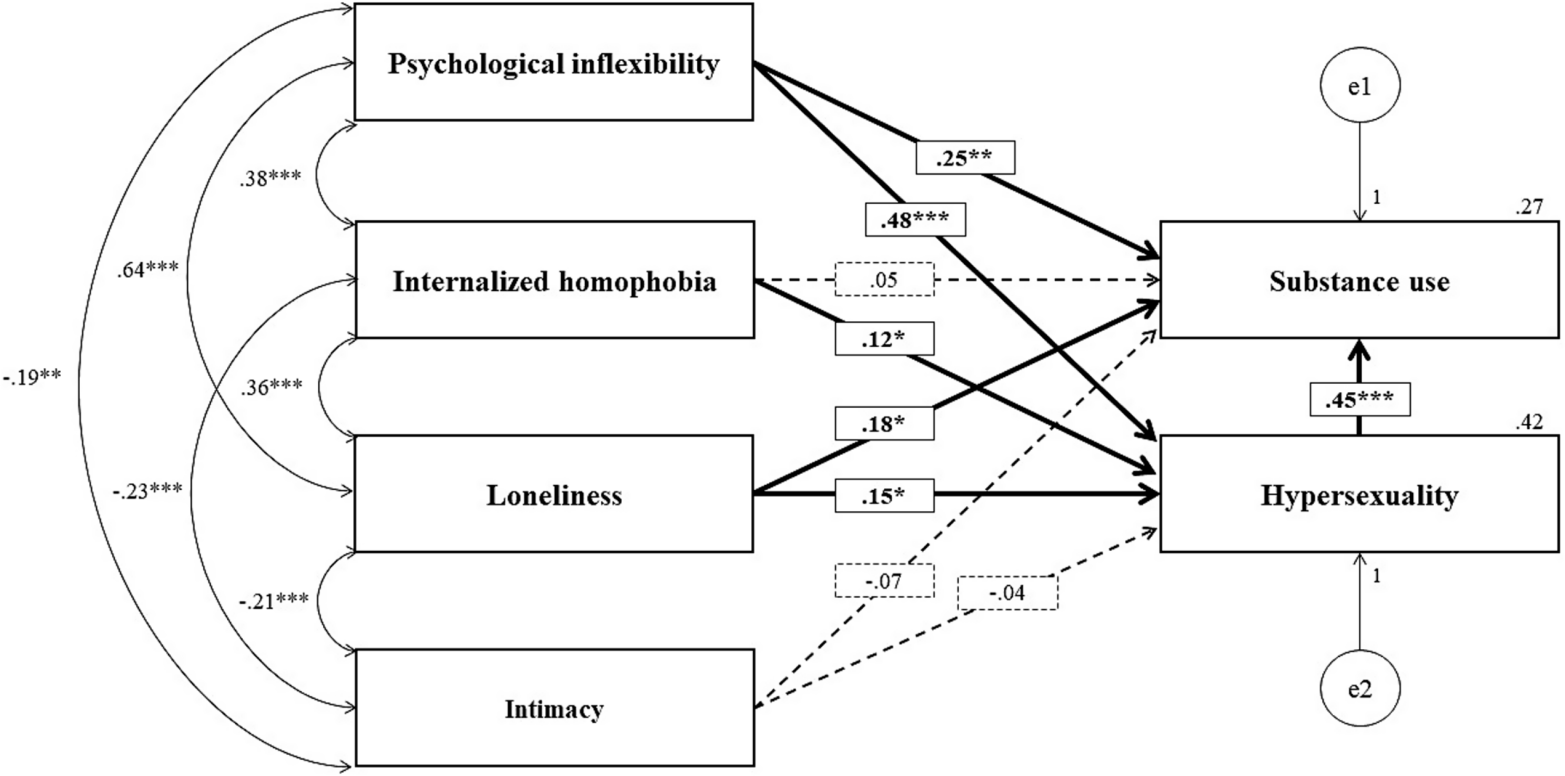
Standardized path coefficients (SE) for structural paths in the model. Standardized path coefficients (β) are displayed for the structural trajectories in the model. Hypothesized but non-significant paths are shown as non-highlighted dotted lines. Significant paths are shown in bold, with significance levels indicated as follows: ***p < 0.001, **p < 0.01, *p < 0.05. Boxes on the left represent exogenous (independent) variables, and boxes on the right represent endogenous (dependent) variables. Unidirectional arrows indicate predictive relationships, and bidirectional arrows between exogenous variables indicate correlations.

Although research on chemsex has grown in recent years, most studies have relied on bivariate associations or multiple regression analyses (Hibbert et al., 2019; Sewell et al., 2018). However, the interrelations among PI, loneliness, intimacy difficulties, internalized homophobia, hypersexuality, and substance use suggest a complex network of interactions that warrants more integrative theoretical and methodological approaches. Structural equation modeling (SEM), particularly path analysis, provides a robust framework for examining these relationships, allowing for the estimation of both direct and indirect effects, as well as the overall fit of the proposed model. This analytical approach aligns well with the principles of contextual behavioral science, as it facilitates a functional understanding of the psychological processes underlying maladaptive behaviors such as problematic chemsex. Moreover, it generates clinically relevant evidence to inform the development of process-based interventions, especially those aimed at enhancing psychological flexibility. Accordingly, the present study aims to investigate the contributions of PI, loneliness, intimacy difficulties, and internalized homophobia to substance use and hypersexuality. These two behaviors were selected as key outcome variables due to their strong theoretical and empirical associations with chemsex (Esteban et al., 2024; Tomkins et al., 2019). Although chemsex was not directly assessed with a single global indicator, we modeled “chemsex vulnerability” through two theoretically proximal behaviors—sexualized substance use and hypersexuality—because (a) there is no widely used, Spanish-validated, single-scale measure capturing the heterogeneity of chemsex practices across contexts and substances; (b) direct, single-item questions (e.g., “Do you practice chemsex?”) risk misclassification due to varying lay definitions; and (c) a process-based approach prioritizes functionally relevant behavioral dimensions over labels. Accordingly, we used validated instruments indexing sexualized drug use and hypersexual behavior as convergent proxies of vulnerability to problematic chemsex engagement. We acknowledge that this indirect operationalization restricts external validity and we expand on its implications in the Limitations section. As illustrated in Figure 1, the hypothesized path model includes both direct and mediated effects from dispositional psychological variables to these behavioral outcomes.

Guided by Contextual Behavioral Science (CBS) and Minority Stress Theory (MST), we specified a process-based path model that links distal contextual stressors to proximal regulatory processes and, in turn, to chemsex-related behaviors. In CBS, psychological inflexibility (PI) is an upstream, transdiagnostic process that fosters experiential avoidance and short-term reinforced patterns (e.g., drug-facilitated sex, compulsive sexual behavior) at the expense of values-congruent living. In MST, internalized homophobia and chronic loneliness (social disconnection) operate as minority-stress correlates that increase shame, hypervigilance, and identity threat, thereby heightening reliance on avoidant coping. Within this integrated framework, hypersexuality is conceptualized as a proximal behavioral response—often serving as a form of experiential avoidance—that may mediate the effects of PI and loneliness on sexualized substance use. Intimacy skills are also included as a potential protective factor, hypothesized to exhibit inverse associations with both outcomes.

This theory-driven mapping leads to the following directional paths in our SEM: (a) direct effects from PI, loneliness, internalized homophobia, and intimacy to hypersexuality and to substance use; (b) indirect effects via hypersexuality from PI and loneliness to substance use (process-based mechanisms); and (c) covariances among exogenous variables, acknowledging their shared minority-stress ecology. SEM is particularly suited to a process-based approach because it simultaneously estimates direct and mediated paths, quantifies overall model fit, and tests whether the data are consistent with the functional architecture posited by CBS and MST. Thus, SEM advances a theory-coherent account of chemsex-related behavior beyond bivariate associations.

In line with this theory-driven specification, directional hypotheses and indirect effects were tested using bias-corrected bootstrap confidence intervals to evaluate the process-based mechanisms linking exogenous variables to sexualized substance use via hypersexuality. Grounded in CBS and MST, and although no analyses were preregistered, we articulated *a priori*, theory-informed expectations: higher psychological inflexibility would be associated with higher hypersexuality (H1a) and higher substance use (H1b), with an indirect pathway from inflexibility to substance use through hypersexuality (H1c); higher loneliness would be linked to greater hypersexuality (H2a) and substance use (H2b), with an indirect effect via hypersexuality (H2c); higher internalized homophobia would predict greater hypersexuality (H3a) and possibly substance use (H3b), with an exploratory indirect pathway via hypersexuality—and, tentatively, via the sequential process inflexibility → hypersexuality (H3c)—consistent with MST and CBS principles; and stronger intimacy skills were expected to show negative associations with both hypersexuality (H4a) and substance use (H4b). Consistent with a minority-stress ecology, all exogenous variables (PI, loneliness, internalized homophobia, and intimacy) were allowed to covary.

## 2 Method

### 2.1 Design

We conducted a cross-sectional observational study employing a descriptive-correlational design. An online survey was administered to a general Spanish-speaking population sample to investigate the contribution of psychological variables—namely psychological inflexibility, loneliness, intimacy, and internalized homophobia—to hypersexuality and substance use.

### 2.2 Inclusion criteria and participants

The following inclusion criteria were established for participation in the survey, while no exclusion criteria were specified. Participants were eligible if they: (1) were 18 years of age or older; (2) were Spanish-speaking; (3) had used psychoactive substances to facilitate, maintain, and/or enhance sexual encounters within the past year; and (4) provided informed consent.

The final sample comprised *N* = 252 participants, ranging in age from 18 to 68 years (*M* = 37.89, SD = 9.26). The majority identified as male (98%) and gay (95.2%), held Spanish nationality (79.4%), and resided in Madrid (61.1%). Additionally, 63.5% were single, 68.7% had completed university-level education, and 79% were currently employed. For a detailed overview of the sample’s sociodemographic characteristics (see Table 1).

**Table 1:** Sample characteristics.

Variable	Values	Frequency	Percentage
Gender identity	Male	247	98%
	Non-binary person	5	2%
Sexual orientation	Gay	240	95.2%
	Bisexual	12	4.8%
Country of origin	Spain	200	79.4%
	Latin America	39	15.5%
	Other	13	5.2%
Region of residence	Madrid	154	61.1%
	Andalusia	30	11.9%
	Catalonia	19	7.5%
	Canary Islands	8	3.2%
	Castilla-La Mancha	8	3.2%
	Other	33	13.1%
Relationship status	Single	160	63.5%
	Open relationship	59	23.4%
	Monogamous relationship	33	13.1%
Educational level	Postgraduate degree	97	38.5%
	Undergraduate degree	76	30.2%
	Vocational training	42	16.7%
	Secondary education	32	12.7%
	Primary education	5	1.9%
Employment status	Employed	199	79%
	Unemployed	38	15.1%
	Student	12	4.8%
	Retired	3	1.2%

N = 252.

Recommendations for structural equation modeling (SEM) suggest a minimum sample size of 200 cases or at least 5–10 times the number of estimated parameters (Bentler and Chou, 1987). The proposed path analysis model includes approximately 21 estimated parameters, encompassing direct effects, residual variances, and covariances among exogenous variables. With an analytic sample of *N* = 252, the study satisfies these recommendations, ensuring adequate statistical power for SEM estimation.

### 2.3 Procedure

An anonymous online survey was developed using the Google Forms platform. The study was conducted in Spain between December 2023 and July 2024, during which time the survey remained open for recruitment. The survey included an introductory section outlining the objectives of the study and the purpose of data collection, followed by an informed consent statement and the self-report questionnaires described in the previous section.

Prior to dissemination, a pilot version of the survey was administered to two individuals who met the inclusion criteria, as well as to a clinical psychologist with expertise in the subject matter. Feedback from these reviewers was used to refine items that were confusing, unclear, or potentially stigmatizing. Minor modifications were implemented, primarily involving the addition of clarifying examples in parentheses to enhance item comprehension.

Participants were recruited through non-probabilistic convenience sampling, following approval from the Research Committee of the European University of Madrid (ID: 2023-324) and the Research Ethics Committee of the Hospital of Getafe (ID: 23.244). The study also received funding from European University of Madrid (ID: 2024/UEM04). Recruitment was carried out in collaboration with the NGO, using incidental dissemination and a snowball sampling strategy—an approach recommended for research involving potentially sensitive topics (Smith, 2013).

To initiate the recruitment phase, a social media influencer and actor (Avelino Piedad, @mravelain on social media) contributed by recording a video encouraging his audience to participate in the study. This video was also shared through the official accounts of the NGO Apoyo Positivo and is available at https://www.instagram.com/p/C3-BWtLtg52/?hl=es.

Participation in the study was entirely voluntary. Anonymity and confidentiality were ensured throughout the research process. Respondents were instructed to complete the self-administered survey in a private, distraction-free setting. No identifying information was collected at any stage. To minimize risks associated with a sensitive topic, the survey was fully anonymous (no IP or contact data stored) and delivered via encrypted transmission. Participants viewed a content warning describing potentially distressing material and could skip any item or withdraw at any time without penalty. Data were stored on password-protected drives accessible only to the research team and reported in aggregate to prevent re-identification.

### 2.4 Measures

#### 2.4.1 Sociodemographic information

The survey collected a range of sociodemographic data, including participants’ age, gender identity, sexual orientation, country of origin, region of residence, relationship status, educational attainment, and employment status.

#### 2.4.2 Variables related to sexual health and chemsex practices

The survey included a series of items designed to characterize participants’ chemsex-related behaviors and sexual health profiles. These items assessed: frequency of barrier protection use during sexual activity; use of pre-exposure prophylaxis (PrEP); number and types of STIs diagnosed in the past year; HIV serostatus and date of the most recent HIV test; frequency of attendance at parties, venues, or gatherings involving sexual activity with multiple partners in the past year; time since last satisfying sexual experience without substance use; typical sexual contexts in which substances are used; predominant methods of contacting sexual partners; primary motivations for substance use in sexual settings; frequency of substance use during sex in the past year; types of substances used in sexual contexts; concerns regarding the consequences of substance use; and efforts to reduce or eliminate substance use in sexual situations.

#### 2.4.3 Hypersexuality

Assessed using the Spanish version of the *Hypersexual Behavior Inventory* (HBI; Ballester-Arnal et al., 2013), a 19-item scale rated on a 5-point Likert scale. Scores ≥53 indicate hypersexuality (García-Barba et al., 2020). The scale shows high reliability (*α* = 0.89–0.96) and was treated as an endogenous variable. Consistent with ICD-11 guidance, HBI scores are used as a dimensional index of hypersexual behavior and not as a standalone diagnostic criterion for CSBD.

#### 2.4.4 Substance use

Assessed using the Spanish version of the *Drug Abuse Screening Test* (DAST-10; Pérez Gálvez et al., 2010), a 10-item Yes/No questionnaire. Scores ≥3 indicate problematic substance use. The scale has high internal consistency (*α* = 0.89) and was treated as an endogenous variable.

#### 2.4.5 Psychological inflexibility

Measured using the Spanish version of the *Acceptance and Action Questionnaire-II* (AAQ-II; Ruiz et al., 2013), a 7-item scale rated on a 7-point Likert scale. Higher scores reflect greater PI. The scale has shown acceptable internal consistency (*α* = 0.74) and was treated as an exogenous variable.

#### 2.4.6 Internalized homophobia

Assessed using the Spanish version of the *Short Internalized Homonegativity Scale* (SIHS; Morell-Mengual et al., 2017), a 13-item Likert scale. Higher scores indicate greater internalized homophobia. The scale has demonstrated high reliability (*α* = 0.80) and was treated as an exogenous variable.

#### 2.4.7 Loneliness

Assessed using the Spanish adaptation of the *Social and Emotional Loneliness Scale for Adults* (SESLA-S; Yárnoz-Yaben, 2008), a 15-item Likert scale. Higher scores indicate greater loneliness. The scale comprises three subscales: social (*α* = 0.71), family (*α* = 0.83), and romantic (*α* = 0.84) loneliness. It was treated as an exogenous variable.

#### 2.4.8 Intimacy

Evaluated using the Spanish version of the *Awareness, Courage, and Responsiveness Scale* (ACRS; Ortiz-Fune et al., 2021), a 24-item self-report measure rated on a 7-point Likert scale. Higher scores reflect stronger intimacy-related skills. The scale has demonstrated excellent reliability (*α* = 0.87–0.91) and was treated as an exogenous variable.

### 2.5 Data analysis

Data were initially compiled using Microsoft Excel and subsequently analyzed with IBM SPSS Statistics (version 27) and AMOS (version 23) for structural equation modeling. To examine the distribution of continuous variables, normality tests were conducted. The Kolmogorov–Smirnov test—appropriate for samples exceeding 50 cases—was applied, along with analyses of skewness and kurtosis. Additionally, frequency histograms were visually inspected to guide the selection of appropriate statistical procedures.

Internal consistency of the questionnaires was assessed using Cronbach’s alpha coefficient, which evaluates the reliability of psychometric instruments. Values range from 0 to 1, with higher scores indicating greater internal consistency. A threshold of *α* ≥ 0.70 was considered acceptable. As the assumption of normality was not met for any of the continuous variables, nonparametric tests were used for inferential analyses. Specifically, Spearman’s rank correlation coefficient was employed to examine associations between continuous variables.

Finally, a structural equation modeling (SEM) approach—specifically path analysis—was employed to examine the hypothesized relationships among the study variables. SEM is a multivariate statistical technique that enables the estimation of multiple interrelated dependencies within a theoretically specified model. In this study, all variables were treated as observed and entered into the model as composite scores derived from validated instruments.

Path analysis was conducted using full information maximum likelihood (FIML) estimation to assess both direct and indirect effects among variables. The model examined associations between the exogenous (independent) variables—psychological inflexibility, internalized homophobia, loneliness, and intimacy—and the endogenous (dependent) variables of hypersexuality and substance use. Model fit was evaluated using multiple indices, including the chi-square test (*χ*^2^), comparative fit index (CFI), root mean square error of approximation (RMSEA), and standardized root mean square residual (SRMR). Conservative cutoff values were applied to determine acceptable model fit: CFI ≥ 0.95, RMSEA ≤ 0.06, and SRMR ≤ 0.08 (Hu and Bentler, 1998). Model estimation and reporting followed the guidelines proposed by Weston and Gore (2006).

## 3 Results

### 3.1 Normality testing and outlier detection

Prior to conducting structural equation modeling (SEM), the dataset was thoroughly screened for missing values, distributional properties, and potential multivariate outliers. No missing data were identified in the final dataset.

To assess the assumptions required for parametric analyses, frequency histograms and the Kolmogorov–Smirnov test were applied to all continuous variables. Visual inspection of the histograms revealed deviations from normality, with varying degrees of skewness and kurtosis. These findings were supported by statistically significant Kolmogorov–Smirnov test results for all variables (*p* < 0.05), indicating non-normal distributions. However, further examination of skewness and kurtosis coefficients revealed no severe violations of normality, as all values remained within acceptable thresholds (absolute skewness < 3; kurtosis < |1|), consistent with the guidelines proposed by Weston and Gore (2006).

Despite the non-normality observed, SEM analysis was conducted, as this approach is robust to moderate violations of normality—particularly in samples exceeding 200 participants. Additionally, the use of maximum likelihood estimation with robust or bootstrap corrections offers further protection against distributional deviations, enhancing the reliability of parameter estimates (Byrne, 2016).

To detect multivariate outliers that could potentially bias model estimates, Mahalanobis distance was calculated. Cases exceeding the critical threshold (*p* < 0.001, based on the number of variables) were excluded from the analysis. As a result, 13 cases were removed, yielding a final analytic sample of *N* = 252 participants. Descriptive statistics and zero-order correlations for the variables included in the model are presented in Table 2. Correlation analyses revealed that PI, loneliness, and internalized homophobia were significantly and positively associated with both hypersexuality and substance use. The strongest correlations were observed between PI and loneliness (*ρ* = 0.612), and between hypersexuality and PI (*ρ* = 0.595), followed by the association between hypersexuality and substance use (*ρ* = 0.512). In contrast, intimacy exhibited weak but statistically significant negative correlations with all variables.

**Table 2:** Descriptive analysis and Spearman’s rank correlation coefficients.

Measure (scale)	1	2	3	4	5	6
1. Psychological inflexibility (AAQ-II)	–					
2. Hypersexuality (HBI)	0.595**	–				
3. Substance use (DAST-10)	0.360**	0.512**	–			
4. Internalized homophobia (SIHS)	0.383**	0.349**	0.196**	–		
5. Loneliness (SESLA-S)	0.612**	0.487**	0.325**	0.359**	–	
6. Intimacy (ACRS)	−0.227**	−0.211**	−0.144*	−0.260**	−0.239**	–
*M*	26.92	49.15	5.09	29.51	55.15	135.96
SD	11.94	19.44	2.27	9.31	18.52	18.69
Mdn	28	49	5	28	55	138
IQR	20.5	34	4	14	26.5	27

*N* = 252; *p < 0.05, **p < 0.01; M, mean; SD, standard deviation; Mdn, median; IQR, interquartile range.

### 3.2 Reliability analysis

The internal consistency of the scales included in the model was assessed using Cronbach’s alpha coefficients. All instruments demonstrated adequate reliability, with alpha values exceeding the recommended threshold of 0.70. Particularly high internal consistency was observed for the PI scale (AAQ-II, *α* = 0.928), the hypersexuality scale (HBI, *α* = 0.956), and the intimacy skills scale (ACRS, α = 0.919), indicating strong item cohesion. The remaining measures also showed satisfactory reliability: substance use (DAST-10, *α* = 0.729), internalized homophobia (SIHS, α = 0.805), and loneliness (SESLA-S, *α* = 0.843).

### 3.3 Descriptive analysis

A descriptive profile of participants’ chemsex-related behaviors and sexual health practices was examined to contextualize the observed psychological patterns. Regarding protective behaviors, 44.8% of participants (*n* = 113) reported never using condoms during sexual intercourse, while 27.4% (*n* = 69) indicated rare use. Only 7.5% (*n* = 19) reported consistent use of barrier protection. In terms of biomedical prevention, 62.7% (*n* = 158) were not currently using pre-exposure prophylaxis (PrEP), whereas 37.3% (*n* = 94) reported active PrEP use. The mean number of STIs diagnosed in the past year was 1.35 (SD = 1.74). The most frequently reported STIs were gonorrhea (34.1%, *n* = 86), syphilis (31.4%, *n* = 79), and chlamydia (27.4%, *n* = 69). Regarding HIV status, 56.8% (*n* = 143) of participants were HIV-negative, 39.7% (*n* = 100) were HIV-positive with an undetectable viral load, and 1.2% (*n* = 3) were HIV-positive with a detectable viral load. As for HIV testing, 19% (*n* = 48) had been tested within the past month, 23.8% (*n* = 60) within the past 1–3 months, and 10.3% (*n* = 26) had not been tested in over a year.

The frequency of attendance at chemsex-related group events varied among participants: 37.5% (*n* = 94) reported attending one to three times per month, while 1.1% (*n* = 3) attended more than three times per week. Regarding substance use during sexual activity over the past year, 13.1% (*n* = 33) reported always using drugs in sexual contexts, 38.1% (*n* = 96) reported frequent use, 31.7% (*n* = 80) occasional use, and 12.3% (*n* = 31) rare use. Participants reported a wide range of substances used in chemsex contexts. The most frequently consumed were, in descending order: poppers, mephedrone, sexual performance-enhancing drugs (sildenafil), alcohol, GHB/GBL, and methamphetamine. Notably, 29% (*n* = 73) had engaged in satisfying sex without substances within the past week, 23.8% (*n* = 60) within the past month, and 21.4% (*n* = 54) within the past 6 months. Chemsex most commonly occurred in group sexual settings, with 78.2% (*n* = 197) reporting substance use during sessions, orgies, or similar gatherings. Mobile dating apps were the predominant means of arranging chemsex encounters, used by 84.9% (*n* = 214) of participants.

Participants reported a variety of motivations for engaging in chemsex. The most frequently cited reasons were enhancing pleasure and sensations (71%, *n* = 179), increasing desire and arousal (66.6%, *n* = 168), and feeling more uninhibited (56%, *n* = 141). Additional motivations included sustaining energy (38.1%, *n* = 96), emotional escape (35.7%, *n* = 90), curiosity and experimentation (34.9%, *n* = 88), improving sexual performance (34.1%, *n* = 86), overcoming insecurity (33.3%, *n* = 84), and engaging in practices they would otherwise avoid (32.5%, *n* = 82). Social and emotional factors were also reported, such as avoiding loneliness (21%, *n* = 53) and seeking emotional connection with partners (19.1%, *n* = 48).

Concerning the perceived consequences of chemsex, 55.5% (*n* = 140) of participants expressed concern about financial costs, while 51.6% (*n* = 130) reported experiencing prolonged physical and psychological recovery periods. Additionally, 38.5% (*n* = 97) noted a loss of control or perceived dependency. Regarding efforts to reduce chemsex behaviors, 26.6% (*n* = 67) had never attempted to do so, 15.5% (*n* = 39) had tried but were unsuccessful, and 63.1% (*n* = 159) reported successful efforts to reduce the frequency of their participation.

### 3.4 Model estimation: psychological inflexibility, internalized homophobia, loneliness and intimacy factors as predictors of substance use and hypersexuality

#### 3.4.1 Measurement model

The measurement model demonstrated excellent fit to the data. The chi-square test was non-significant (*χ*^2^ = 0.002, *p* = 0.967), indicating minimal discrepancy between the observed and expected covariance matrices. Complementary fit indices further supported the model’s adequacy: CFI = 1.000, RMSEA = 0.000, and SRMR = 0.011—all within commonly accepted thresholds, suggesting a strong and well-specified model fit.

#### 3.4.2 Structural model

As illustrated in Figure 1 and detailed in Table 3, the structural pathways confirmed several of the hypothesized relationships. PI demonstrated a significant direct effect on substance use (*β* = 0.251, *p* < 0.01) and a strong direct effect on hypersexuality (*β* = 0.481, *p* < 0.001), suggesting that it may function as a key upstream variable influencing both behavioral outcomes. Furthermore, hypersexuality showed the strongest association with substance use (*β* = 0.445, *p* < 0.001), reinforcing its role as a central mediating variable.

**Table 3:** Measurement model.

Endogenous variable	Predictor	Unstandardized	Standardized			
		*B*	*β*	95% CI	SE	*p*-value
Substance use (DAST-10)	Psychological inflexibility (AAQ-II)	0.045	0.251	[0.012, 0.078]	0.017	<0.01
Substance use (DAST-10)	Internalized homophobia (SIHS)	0.013	0.053	[−0.018, 0.044]	0.016	0.415
Substance use (DAST-10)	Loneliness (SESLA-S)	0.021	0.179	[0.003, 0.039]	0.009	0.020
Substance use (DAST-10)	Intimacy (ACRS)	−0.008	−0.065	[−0.022, 0.006]	0.007	0.278
Substance use (DAST-10)	Hypersexuality (HBI)	0.052	0.445	[0.036, 0.068]	0.008	<0.001
Hypersexuality (HBI)	Psychological inflexibility (AAQ-II)	0.782	0.481	[0.578, 0.986]	0.104	<0.001
Hypersexuality (HBI)	Internalized homophobia (SIHS)	0.26	0.125	[0.042, 0.478]	0.111	0.019
Hypersexuality (HBI)	Loneliness (SESLA-S)	0.155	0.148	[0.026, 0.284]	0.066	0.020
Hypersexuality (HBI)	Intimacy (ACRS)	−0.046	−0.044	[−0.148, 0.056]	0.052	0.372

B, unstandardized regression coefficient; β, standardized regression coefficient; 95% CI, 95% confidence interval; SE, standard error; p, significance value of the test.

Loneliness also had a significant path coefficient toward hypersexuality (*β* = 0.148, *p* = 0.020), as well as a modest but significant effect on substance use (*β* = 0.179, *p* = 0.020), indicating its relevance across both outcomes. Internalized homophobia was positively associated with higher levels of hypersexuality (*β* = 0.125, *p* = 0.019); however, its direct effect on substance use was not significant (*β* = 0.053, *p* = 0.415). The structural paths from intimacy to both hypersexuality (*β* = −0.044, *p* = 0.372) and substance use (*β* = −0.065, *p* = 0.278) were not statistically significant. Given the non-significant paths from intimacy to both outcomes and the absence of model fit deterioration when constraining these paths to zero, intimacy was retained as a covariate in sensitivity checks but excluded from the final parsimonious model.

#### 3.4.3 Indirect effects

To further examine the mechanisms underlying these associations, three indirect effects were tested using 1,000 bootstrapped samples with bias-corrected confidence intervals. As shown in Table 4, the results indicated that PI was indirectly associated with substance use through its influence on hypersexuality (*β* = 0.22, 95% CI [0.10, 0.33], *p* = 0.004). Similarly, loneliness predicted substance use indirectly via hypersexuality (*β* = 0.08, 95% CI [0.01, 0.17], *p* = 0.019). In contrast, the indirect effect of internalized homophobia on substance use through hypersexuality did not reach statistical significance (*β* = 0.05, 95% CI [−0.01, 0.12], *p* = 0.092).

**Table 4:** Indirect effects analyses.

Paths	Standardized indirect effect		Bootstrap estimate			95% CI bootstrap bias corrected
	*β*	SE	*β*	SE	*p*-value	
Psychological inflexibility (AAQ-II) → Hypersexuality (HBI) → Substance use (DAST-10)	0.22	0.06	0.22	0.06	0.004	[0.11, 0.35]
Internalized homophobia (SIHS) → Hypersexuality (HBI) → Substance use (DAST-10)	0.05	0.03	0.05	0.03	0.092	[−0.01, 0.12]
Loneliness (SESLA-S) → Hypersexuality (HBI) → Substance use (DAST-10)	0.08	0.04	0.08	0.04	0.019	[0.01, 0.17]

β, standardized regression coefficient; SE, standard error; p, significance value of the test; 95% CI bootstrap bias corrected, 95% confidence interval with bias correction using bootstrapping.

#### 3.4.4 Explained variance

The structural model accounted for a moderate proportion of variance in the outcome variables. Specifically, the set of predictors explained 27.2% of the variance in substance use (*R*^2^ = 0.272) and 42.1% in hypersexuality (*R*^2^ = 0.421), suggesting that the model provides a meaningful, albeit partial, explanatory framework for these chemsex-related behaviors.

## 4 Discussion

The present study aimed to examine the psychological processes underlying hypersexuality and sexualized substance use among gay and bisexual men who engage in chemsex, adopting a contextual behavioral perspective. Consistent with the proposed model, PI emerged as a central dispositional factor, exerting both direct and indirect effects on substance use through hypersexuality. These findings underscore the relevance of PI as a transdiagnostic process underlying maladaptive coping strategies in the context of chemsex, and they align with previous research highlighting its predictive role in compulsive sexual behavior and substance-related problems (Ortega-Otero et al., 2023; Krotter et al., 2024).

Beyond the structural associations examined in this study, the descriptive findings provide valuable contextual insights into the sexual health and chemsex-related behaviors of the sample. A considerable proportion of participants reported inconsistent condom use, limited engagement with biomedical prevention strategies such as pre-exposure prophylaxis (PrEP), and a high incidence of STIs within the past year—particularly gonorrhea, syphilis, and chlamydia. These patterns highlight significant vulnerabilities in sexual health among individuals engaging chemsex.

Importantly, the most frequently reported motivations for chemsex practice—such as enhancing pleasure, increasing arousal, and alleviating emotional distress—underscore the reinforcing nature of these behaviors and suggest their potential role as mechanisms of emotional regulation. The relatively high proportion of participants who expressed concern about the consequences of chemsex, along with reported efforts to reduce or control their use, reflects an ambivalent relationship with these practices and indicates a clinically relevant window of opportunity for intervention.

PI emerged as the strongest predictor of hypersexuality, supporting the hypothesis that difficulties in regulating aversive internal experiences may lead individuals to engage in compulsive sexual behavior as a form of experiential avoidance. These findings are consistent with previous research (Jaspal, 2020; Montesinos and Ortega, 2022; Rodríguez-Expósito et al., 2024). Moreover, this result aligns with ACT models of behavioral dysregulation, which posit that short-term relief from internal distress (e.g., loneliness, shame, or identity-related conflict) is often prioritized over long-term valued living (Hayes et al., 2006). The observed indirect effect of PI on substance use through hypersexuality further supports a hierarchical model in which hypersexuality functions as a proximal mediator linking PI to chemsex-related substance use. These findings underscore the potential utility of interventions aimed at enhancing emotional acceptance and distress tolerance as mechanisms for reducing reliance on avoidant coping strategies.

Loneliness also demonstrated significant associations with both outcome variables, suggesting that social and emotional disconnection may constitute a key vulnerability factor in chemsex engagement. This finding aligns with prior research reporting elevated levels of loneliness among GBMSM who engage in chemsex (Hung et al., 2023), and supports the notion that drug-facilitated sexual encounters may serve as maladaptive attempts to alleviate unmet intimacy needs (Lafortune et al., 2021; Lunchenkov et al., 2024). Notably, the indirect effect of loneliness on substance use through hypersexuality suggests that, for some individuals, the emotional void may be filled through compulsive sexual behavior, which in turn reinforces substance use patterns. However, the perceived intimacy within chemsex contexts often fails to translate into enduring relational bonds, potentially perpetuating cycles of loneliness and reinforcing problematic behaviors (Moreno-Gámez et al., 2022; Mundy et al., 2025).

Internalized homophobia significantly predicted hypersexuality but did not exhibit a direct effect on substance use, and its indirect effect via hypersexuality did not reach statistical significance. These findings offer partial support for the minority stress hypothesis (Meyer, 2003), suggesting that internalized stigma may contribute to dysregulated sexual behavior, potentially through mechanisms such as shame, self-silencing, and sexual identity avoidance (Brumfield and Dahlenburg, 2025). However, the weaker-than-expected associations may reflect the partial mediation of this process by psychological inflexibility—a variable not tested as a serial mediator in the current model but one that warrants further investigation in future research.

Contrary to initial expectations, the intimacy variable did not exhibit significant associations with either hypersexuality or substance use. This finding may suggest that the capacity for emotional closeness, as measured in this study, does not directly translate into protective behaviors within chemsex contexts, or that its effects are overshadowed by more salient predictors such as loneliness and PI. An alternative explanation is that intimacy-related deficits may play a more prominent role in the maintenance, rather than the initiation, of chemsex practices—particularly when repeated failures to establish meaningful connections reinforce cycles of experiential avoidance. The null effects of intimacy may reflect (a) construct mismatch—our ACRS index captures general intimacy skills rather than sexual/romantic intimacy in chemsex-specific contexts; (b) suppression by stronger, proximal predictors (PI, loneliness); and/or (c) stage-specific influence (maintenance vs. initiation); (d) potential construct validity issues, insofar as participants may have tended to evaluate themselves more favorably than their actual performance in intimacy-related situations, thereby introducing self-report biases. Future studies should test intimacy facets more proximal to sexual contexts (e.g., comfort with sober intimacy, attachment-related avoidance/anxiety) and evaluate potential non-linear or moderated effects (e.g., by minority stress), while also incorporating multimethod measures to reduce self-report bias.

The strong interconnection between hypersexuality and substance use underscores their mutual reinforcement. This finding is consistent with clinical observations indicating that chemsex often establishes a highly reinforcing behavioral loop, wherein the pharmacological effects of substances enhance sexual desire and disinhibition, while sexual compulsivity drives the use of substances to sustain or intensify sexual encounters (Nimbi et al., 2021; Irfan et al., 2023). Consequently, these behaviors appear to co-occur, rather than represent entirely independent phenomena, highlighting the need for integrated interventions that address both dimensions simultaneously.

From a contextual behavioral science perspective, these findings underscore the functional role of chemsex as a behavior which may be maintained by short-term experiential avoidance, emotion regulation deficits, and social isolation. While chemsex may initially serve to suppress or escape internal distress (e.g., loneliness, shame, negative self-perceptions), it often leads to further psychological dysregulation and detachment from valued living. Notably, the model accounted for 42.1% of the variance in hypersexuality and 27.2% in substance use, indicating moderate explanatory power and highlighting the relevance of additional factors—such as trauma history, impulsivity, or community-level influences—that warrant inclusion in future models (Wéry et al., 2018; Jepsen et al., 2023; Belfiore et al., 2024). Extending the model with theoretically relevant processes—impulsivity/urgency, trauma history and post-traumatic symptoms, and minority-stress moderators (e.g., anticipated rejection, concealment)—may increase explained variance and clarify pathways to sexualized substance use.

From a minority stress perspective, these findings reinforce the notion that chemsex is not merely a recreational activity but can become a maladaptive coping strategy rooted in stigma, social disconnection, and emotional dysregulation (Meyer, 2003; Pachankis et al., 2015). Accordingly, future interventions must be culturally affirming and socially grounded, addressing not only individual psychological processes but also community-level determinants such as access to safe spaces, supportive social networks, and the affirmation of sexual identity.

The clinical implications of these findings include the potential utility of ACT and other process-based interventions aimed at enhancing psychological flexibility. Targeting PI may yield cascading benefits across multiple domains implicated in chemsex, including hypersexuality, substance use, and minority stress-related constructs. ACT-based interventions that incorporate components such as cognitive defusion, emotional openness, and values-based action may be particularly effective in helping individuals cultivate healthier relationships with sexuality, interpersonal connection, and emotional discomfort, as demonstrated in previous studies (Montesinos and Ortega, 2022; Strika-Bruneau et al., 2023; Montesinos et al., 2024). These findings also align with emerging approaches such as Mindfulness-Based Queer Resilience (MBQR; Sun et al., 2022), which integrate minority stress awareness into skills training for LGBTIQ+ individuals.

Findings support process-based intervention targets consistent with ACT model. For psychological inflexibility and experiential avoidance: cognitive defusion (e.g., “the hungry tiger metaphor” with language cues for chemsex triggers), acceptance and distress tolerance for shame/loneliness, and flexible present-moment attention skills during high-risk sexual contexts. For values-based living: clarification of sexual/relational health values, committed actions (e.g., pre-planning substance-free intimacy, safer-sex routines), and reinforcement of prosocial alternatives. For minority stress: perspective-taking and self-compassion practices; values-consistent boundary setting; stigma-related defusion. Delivery can be tailored to (i) individual formats (functional analysis of personal triggers; graded exposure to sober intimacy), (ii) group formats (psychoeducation, skills rehearsal, values work, peer modeling), and (iii) community-based services (drop-in groups, integration with sexual-health/PrEP clinics, app-based boosters for cravings). Integrating CSBD-informed components (e.g., stimulus control, relapse prevention) with ACT processes may address the reciprocal loop between hypersexuality and sexualized substance use. The pattern of indirect effects (PI → hypersexuality → substance use; loneliness → hypersexuality → substance use) suggests that targeting PI and loneliness may yield downstream benefits on both behaviors.

Specifically, therapeutic efforts could focus on helping individuals disentangle from shame-laden thoughts associated with identity or sexuality, increase their willingness to experience difficult emotions such as loneliness without resorting to avoidance strategies, and clarify personally meaningful values related to intimacy, health, and connection. Group-based formats that integrate experiential exercises and values work may be particularly well-suited for fostering both individual and community-level resilience. Ultimately, the proposed model provides a functional roadmap for developing culturally sensitive, process-based interventions aimed at reducing chemsex vulnerability while enhancing psychological flexibility and well-being in sexual minority populations.

Beyond clinical practice, the present findings have policy-relevant implications. First, inclusive, sex-positive sexual health education that explicitly addresses drug–sex interactions, consent under intoxication, and sober-intimacy skills may reduce reliance on avoidance-driven coping. Second, harm-reduction access—including drug-checking services, pragmatic guidance on GHB/GBL dosing and redosing intervals, stimulant comedown management, and non-punitive post-exposure pathways—should be integrated within sexual health services. Third, culturally sensitive mental health programs (e.g., ACT-based groups targeting psychological inflexibility, shame, and loneliness) embedded in community settings and co-located with PrEP/PEP clinics can lower barriers and capitalize on existing care pathways. Fourth, digital outreach (dating apps, encrypted messaging) can deliver just-in-time education and micro-interventions around high-risk windows (e.g., weekends), aligning with the patterns observed in GBMSM communities. Fifth, provider-level policies—training clinicians in minority-stress–informed, non-judgmental care and ensuring confidentiality—may mitigate stigma, a driver of experiential avoidance. Lastly, city- and clinic-level monitoring systems that pair behavioral indicators (hypersexuality, sexualized substance use) with service utilization can inform resource allocation and evaluate the impact of community interventions over time. These strategies align with a process-based prevention agenda: reduce contextual stressors (stigma, isolation), increase flexible responding (psychological flexibility, values-based action), and ensure pragmatic harm-reduction supports in the ecosystems where chemsex occurs.

### 4.1 Limitations of the study and proposal for future research lines

First, chemsex was operationalized indirectly via validated measures of sexualized substance use and hypersexual behavior. While theoretically justified within a process-based framework, this approach may not fully capture the phenomenology, patterns (e.g., specific substances, routes), and contextual heterogeneity of chemsex; thus, external validity is constrained. Future studies should include direct, validated chemsex measures alongside behavioral indices. Second, the sample predominantly comprised cisgender gay men residing in urban Spain, limiting generalizability to bisexual men, *trans* and non-binary people, and non-urban or non-Spanish-speaking contexts. Third, the intimacy construct was assessed with a broad skills measure (ACRS) that may not index intimacy processes most proximal to chemsex; alternative measures and potential moderators should be tested. Finally, the cross-sectional, self-report design precludes causal inference and is subject to reporting biases; longitudinal and mixed-methods designs are needed.

Finally, while this study offers novel insights, several limitations must be acknowledged. The cross-sectional design precludes causal inference, and the reliance on self-report measures may introduce response biases. In particular, self-report instruments are susceptible to social desirability effects, recall biases, subjective interpretation of item content, and potential response fatigue or inattention. Moreover, the use of an online survey format—while advantageous for accessing hidden or stigmatized populations—limits control over the conditions under which participants completed the questionnaires, potentially affecting the quality and consistency of responses. The sample was primarily composed of cisgender gay men residing in urban areas of Spain, which limits the generalizability of the findings to broader populations. Additionally, chemsex was operationalized indirectly through measures of substance use and hypersexuality, which—although theoretically justified—may not fully capture the complexity and phenomenology of chemsex engagement. Furthermore, other potentially relevant variables, such as impulsivity, trauma history, and clinical symptomatology, were not assessed. Future research should employ longitudinal designs, explore typologies of chemsex users, and develop ACT-based interventions tailored to the specific needs of this population.

## 5 Conclusion

This study offers a novel contribution to the literature by advancing a contextual behavioral model of chemsex vulnerability, identifying key psychological processes underlying hypersexual behavior and substance use among GBMSM. PI emerged as a central transdiagnostic factor, showing both direct and indirect effects on these behaviors, which appear to function as experiential avoidance strategies aimed at mitigating internal distress related to loneliness, stigma, and internalized homophobia. Hypersexuality served as a key mediator between psychological inflexibility and substance use, while loneliness demonstrated both direct and mediated effects on these outcomes. Although difficulties in establishing intimacy did not significantly predict either behavior, the findings support a functional account in which chemsex may be maintained by short-term emotional relief at the expense of long-term well-being. These results underscore the relevance of ACT-based interventions focused on reducing experiential avoidance and fostering more flexible, values-guided behavior among sexual minority populations.

## Data Availability

The raw data supporting the conclusions of this article will be made available by the authors, without undue reservation.
